# Landing System Development Based on Inverse Homography Range Camera Fusion (IHRCF)

**DOI:** 10.3390/s22051870

**Published:** 2022-02-27

**Authors:** Mohammad Sefidgar, Rene Landry

**Affiliations:** LASSENA Laboratory, École de Technologies Supérieure (ÉTS), Montreal, QC H3C 1K3, Canada; renejr.landry@etsmtl.ca

**Keywords:** inverse planar homography, sensor fusion, navigation landing system design, pose estimation

## Abstract

The Unmanned Aerial Vehicle (UAV) is one of the most remarkable inventions of the last 100 years. Much research has been invested in the development of this flying robot. The landing system is one of the more challenging aspects of this system’s development. Artificial Intelligence (AI) has become the preferred technique for landing system development, including reinforcement learning. However, current research is more focused is on system development based on image processing and advanced geometry. A novel calibration based on our previous research had been used to ameliorate the accuracy of the AprilTag pose estimation. With the help of advanced geometry from camera and range sensor data, a process known as Inverse Homography Range Camera Fusion (IHRCF), a pose estimation that outperforms our previous work, is now possible. The range sensor used here is a Time of Flight (ToF) sensor, but the algorithm can be used with any range sensor. First, images are captured by the image acquisition device, a monocular camera. Next, the corners of the landing landmark are detected through AprilTag detection algorithms (ATDA). The pixel correspondence between the image and the range sensor is then calculated via the calibration data. In the succeeding phase, the planar homography between the real-world locations of sensor data and their obtained pixel coordinates is calculated. In the next phase, the pixel coordinates of the AprilTag-detected four corners are transformed by inverse planar homography from pixel coordinates to world coordinates in the camera frame. Finally, knowing the world frame corner points of the AprilTag, rigid body transformation can be used to create the pose data. A CoppeliaSim simulation environment was used to evaluate the IHRCF algorithm, and the test was implemented in real-time Software-in-the-Loop (SIL). The IHRCF algorithm outperformed the AprilTag-only detection approach significantly in both translational and rotational terms. To conclude, the conventional landmark detection algorithm can be ameliorated by incorporating sensor fusion for cameras with lower radial distortion.

## 1. Introduction

Landing system design and UAV descending landmark detection have been the focus of many studies. The prime aim of these efforts is to develop highly accurate and computationally lightweight algorithms to meet the needs of businesses and emergency UAVs that serve in marine environments.

### 1.1. Problem Statement

The main drawback of landing system development research thus far is that it has been centered around less complex landing platforms such as static or minimally fluctuating ship decks. Hence, there is a need to investigate landing surfaces with higher levels of motion complexity, such as a Stewart table, for pose estimation. The work presented here is a new range and camera sensor fusion technique applicable to complex landing tasks that addresses the pose estimation problem of a descending surface by utilizing Inverse Homography Range Camera Fusion (IHRCF). IHRCF operates by first calculating the homography between pixels and world coordinates. Then, by inverse homography, Apriltag pixels are transformed to world coordinates. Next, knowing the real-world coordinates of the AprilTag corners, the rigid body transformation between the camera frame and real-world landmark coordinates is calculated using range sensor data. Finally, depending on the sensor installation and the type of acquisition, which is here dependent on the CoppeliaSim convention of image data acquisition, a transformation step is required. This proposed IHRCF technique was evaluated, and its performance indicates it is a viable algorithm for UAV pose estimation.

### 1.2. Literature Review

The methods found in the literature can be categorized into two main sections: conventional and modern approaches. While modern methods use deep learning and machine learning for positioning, conventional methods use algorithms such as Kalman Filter (KF), Extended Kalman Filter (EKF), Unscented Kalman Filter, and Particle Filter (PF). There are some drawbacks to address the issue of pose estimation using these methods. Regarding modern methods, they utilize black box for modeling the complex system. This significantly increases the computational expenses of the designed system, leading to including a Graphical Processing Units (GPUs) which can handle heavy calculations [1]. In addition, training a deep model requires the availability of large datasets for training which is sometimes impractical or imprecise [2]. Power and memory demands are escalating when deep learning model becomes deeper, resulting in a weighty system design and lowering flight time, in the case of aerial autonomous design [1]. The training process also for the Ultra Deep Neural Network (UDNN) is resource intensive and time consuming [3]. Furthermore, concerning conventional methods, KF is very well known to handle the linear system with high accuracy. However, it cannot be used for the nonlinear system. Hence EKF is used for systems with moderate nonlinearity [4]. EKF can, to some extent, handle system nonlinearity up to the point that the noise statistic such as covariance matrixes of the process noise is accurate, otherwise the estimation can either be inaccurate or diverge [5]. Therefore, it can be inferred that EKF error estimates tend to undervalue state uncertainties [6]. UKF, to a great extent, compensates the deficiencies of the EKF in system nonlinearity, but it still suffers from the fact that rounding error and approximation error become larger and larger when nonlinearity of the system increases [7]. PF was developed to handle even more complex system with very high nonlinearity. The accuracy of PF largely depends on the number of particles, this can boost the computations, leading to the expensive and heavy system design [8]. We developed a sensor fusion technique that can improve AprilTag detection algorithms (ATDA) pose estimation in terms of rotational and translational estimation. The designed system is very lightweight in compared to heavy LIDAR system and generates only four single line range data, which significantly lower the computations.

Sensors used for pose estimation and location include monocular and stereo cameras, Inertial Measurement Units (IMU), Global Positioning System (GPS), and Impulse Radio Ultra-Wideband (IR-UWB). A simple landing system consists of a rope and a tether for landing platform pose estimation. Most conventional methods apply an estimation theory to compensate for signal noise. Two works focused on the vision and inertial measurements-based algorithm almost at the raw-sensor level to conduct EKF sensor fusion leading to a robust and rapid pose estimation [9,10]. A subsequent study employed a camera and an IMU sensor fusion algorithm using EKFs that made use of the more robust features from the camera data for object detection: the speeded-up robust feature (SURF) and random sample consensus (RANSAC). The algorithm proved to be computationally rapid, robust and reliable, and thus can be considered for practical applications [11]. A more nonlinear estimation would be to use an UKF that can better handle the nonlinearity of the system models. Researchers used a variation of the UKF called the Square-root Unscented Kalman Filter (SR_UKF) combined with the concept of homography between the geoinformation of the landing platform and data from vision and IMU fusion sensors. They found that their proposed method could be used for the accurate landing of commercial aircraft in GPS-denied environments and in low visibility [12]. Another work used a small-sized drone to conduct pose estimation, with the camera installed on the ship deck to reduce the size of the drone for test purposes. An even more nonlinear filter, cited as a particle filter, was used to extract the bounding box feature from each frame of the camera. These features are important for sampling, weighting and resampling. A UKF and an Unscented Bingham Filter (UBiF) were used for the translational motion filtering and the rotational motion filtering, respectively. This study showed that the developed system performance satisfied the automatic landing system requirements [13]. What is more, the stereo camera found its place in drone-based localization and navigation of UAVs, as it offers 3D data. Kim and Lee (2003) proposed a system based on the stereo camera, using a Kalman filter to reduce drift from IMU data and making use of Differential GPS (DGPS). They concluded that the technique could provide accurate attitude and position data for GPS-denied environments [14]. Another study focused on stereo vision and inertial navigation data fusion using basic Kalman filtering and featured matching between stereo frames for the aims of takeoff, hovering and landing. The main advantage of this work was that it provided an algorithm independent of the GPS or landmark coordinates for positioning [15]. The fisheye camera is another camera variant, one that furnishes a wider angle of view compared to monocular cameras. The fusion of the features data from the camera and stereo vision provides a system with sufficient accuracy and robustness to successfully predict landings on a moving Unmanned Ground Vehicle (UGV) [16]. Besides, a work by Wang and She (2017) proposed a multi-landmark method for moving rover landing research by utilizing a gimbaled camera instead of a fisheye camera. This approach could prompt the camera’s Field of View (FOV) [17]. Finally, a sensor fusion technique based on image and IMU data has met the challenging task of guiding beyond visual line of sight (BVLOS) by First Person View (FPV) automatic navigational equipment. Estimated poses were amended by adding a term cited as a correction function calculation to the estimated pose [18].

While the above-mentioned conventional techniques applied filters such as the EKF and the UKF, modern research approaches utilize Artificial Intelligence (AI) [19,20,21,22,23]. Powerful Graphical Processing Units now allow for rapid calculations [24,25,26]. Most basic intelligent landing systems use neural networks. At least one uses a neural network trained by fuzzy logic for descending landmark detection [27]. Another study focuses on developing a safe landing detection algorithm from 3D lidar point clouds, aiming to outperform the conventional heuristic methods [28]. Reinforcement learning is a technique that employs a base agent which interacts with the environment through actions. The agent is awarded or penalized for every action it takes. Therefore, the agent is trained until it finds a way to maximize its reward while minimizing its penalty through its experience obtained through its actions [29,30,31]. Another approach investigates deep reinforcement learning based on Deep Q-Networks (DQNs) as a control method to navigate towards the landing landmark [32]. The following sub-sections discuss the settings, installations, and methodologies utilized in this work.

## 2. Proposing Inverse Homography Range Camera Fusion (IHRCF) Methodology

**Figure 1:**
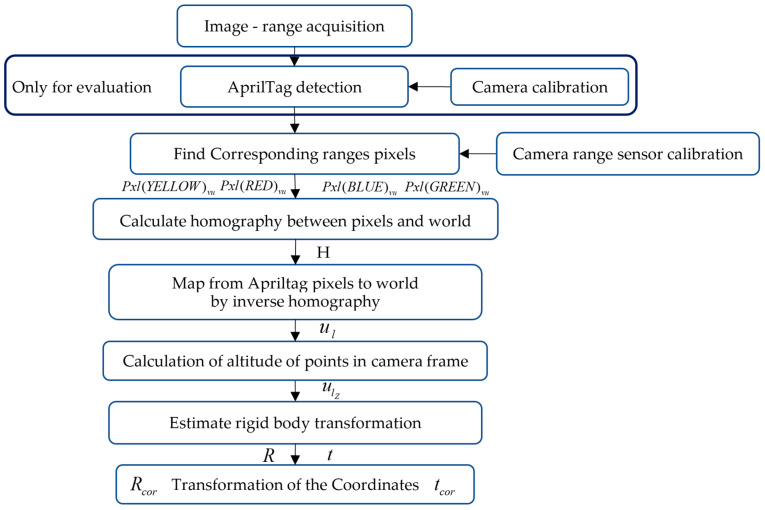
Overview of the IHRCF algorithm and evaluation process.

Figure 1 presents an overview of the developed IHRCF system, including its evaluation process. The landing position of the drone is vertical, and the drone processes data from AprilTag via the proposed algorithm. The IHRCF only requires four-point pixel coordinates and their distances in the real world. Therefore, the algorithm does not require a camera calibration phase. The overview of the technique requires A ToF and Camera calibration technique in advance of the IHRCF to relate distances from ranges sensors to the pixel in the image. The technique starts by collecting images from the camera and detection of four corners of the AprilTag. Knowing world dimensions of the sensors and the touch points in the image, the planar homography is calculated. Since the homography and pixel coordinate of the AprilTag corners are known, *X* and *Y* directions of the landmark can be found using inverse homography in the next step. Then these directions data are used to find altitude of the corner points by the planar surface equation found by 4 ranges sensors. Subsequently, rigid body transformation between found world coordinate of the corners and known dimension of the AprilTag is calculated. In the final stage, the simulator coordinate system is converted to the Cartesian coordinate translations and Euler angles.

**Figure 2:**
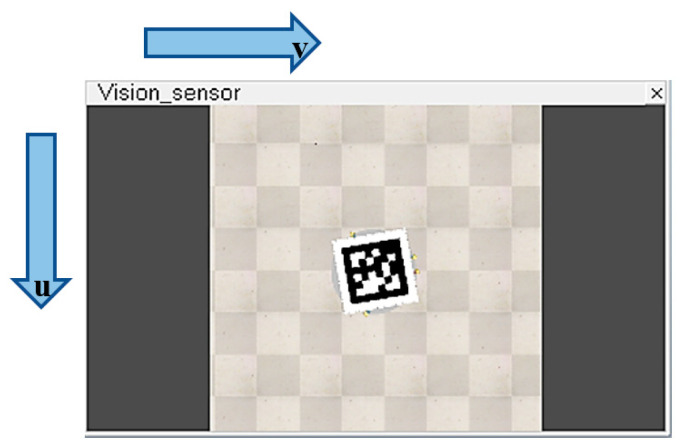
Drone downward camera view of the landing platform-*u* and *v* Pixel coordinate.

**Figure 3:**
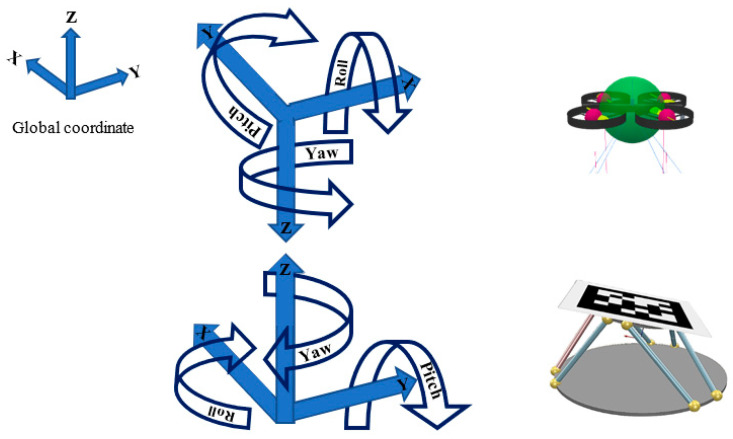
Cartesian coordinates of the landing platform and the designated drone.

**Figure 4:**
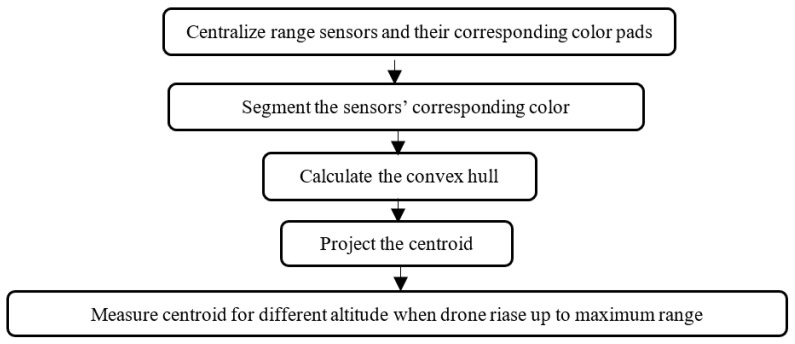
Camera range calibration procedures in each stage.

Figure 2 represents the pixel coordinate of the camera, while Figure 3 and Figure 4 illustrate the Cartesian coordinates of the drone and landing surfaces.

### 2.1. Camera Calibration

Camera calibration is used here in the AprilTag detection algorithm (ATDA) to evaluate the performances of the proposed IHRCF approach. The camera calibration process is the attempt to find the camera’s mathematical model. Generally, a camera has the following relations:

(1) $$s\left[\begin{array}{c} u\\ v\\ 1 \end{array}\right]=\left[\begin{array}{ccc} f_{x} & 0 & c_{x}\\ 0 & f_{y} & c_{y}\\ 0 & 0 & 1 \end{array}\right]\left[\begin{array}{cccc} r_{11} & r_{12} & r_{13} & t_{x}\\ r_{21} & r_{22} & r_{23} & t_{y}\\ r_{31} & r_{32} & r_{33} & t_{z} \end{array}\right]\left[\begin{array}{c} X_{w}\\ Y_{w}\\ Z_{w}\\ 1 \end{array}\right]$$

where *f_x_*, *f_y_*, *c_x_*, and *c_y_* are the camera focal lengths and camera principal points, respectively, in the *x* and *y* directions. The matrix that contains the focal lengths and principal points is called the intrinsic parameter of the camera. Moreover, *r_ij_* and *t_x,_ t_y_*_,_ and *t_z_* are the camera rotations and translations. The matrix that includes the rotation elements and the translation is called the extrinsic parameter of the camera. Equation (1) can be used to calculate the pixel coordinates of any point in the world if the *X*, *Y*, and *Z* coordinates of that point in the world are known.

The standard camera calibration method follows the steps listed below:

- Obtain the chessboard images with different rotations and translations in the camera frame.
- Calculate the grayscale image from the acquired chessboard images.
- Apply the corner detection algorithm; and
- Use the real-world camera position to calculate the homography, using the chessboard’s size, which has squares of 2.5 mm and 7 × 9 corners [33,34,35,36].

### 2.2. Camera Range Sensor Calibration

In the simulation, the drone is commanded to increase the altitude, stop and take a picture in 120 stages every 3 s, allowing it to stabilize after each altitude increase, up to the maximum sensor range, refer to Figure 4.

**Figure 5:**
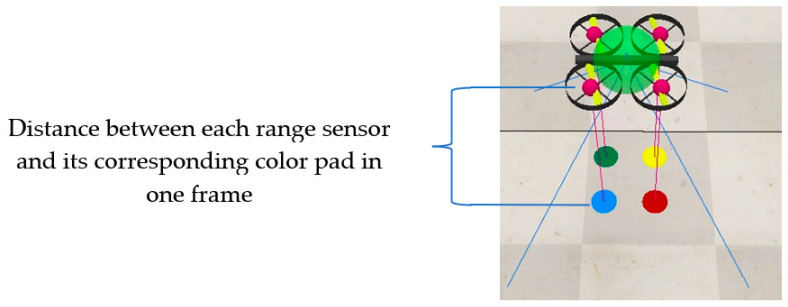
Range sensors’ touch points centralization with their corresponding color pads.

The motivation for camera range sensor calibration is to find the coordinates of the pixels at different altitudes. Therefore, four circular calibration pads, each of a different color: blue, green, yellow, and red, are chosen and centralized with each sensor. In this attempt the blue, green, yellow, and red pads are centralized with sensors ps_1_, ps_2_, ps_3_, and ps_4_ touch points, respectively. Figure 5 illustrates the relative location of range sensors and their allocated color pads only for one frame.

In the next phase, images are collected via the downward camera and each color pad is segmented by the conversion of images from RGB to YCBCR color format using Equation (2) [37]:

(2) $$\left[\begin{array}{c} 601_{Y}\\ 219^{\prime }\\ C_{B}\\ C_{R} \end{array}\right]=\left[\begin{array}{c} 16\\ 128\\ 128 \end{array}\right]+\left[\begin{array}{ccc} 65.481 & 128.553 & 24.966\\ -37.797 & -74.203 & 112\\ 112 & -93.786 & -18.214 \end{array}\right]\cdot {\left[\begin{array}{l} R\\ G\\ B \end{array}\right]}^{T}$$

**Figure 6:**
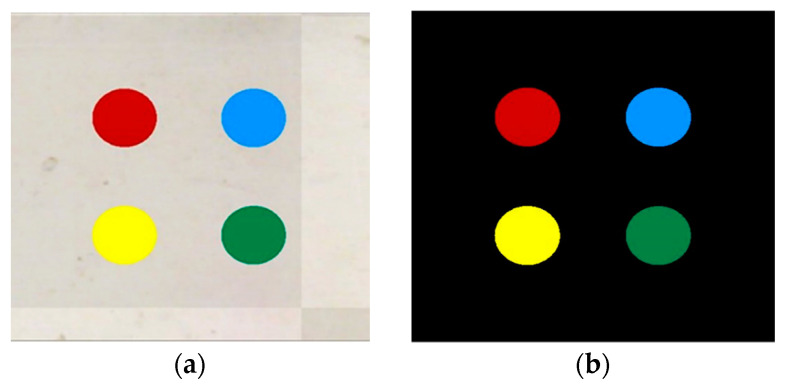
Color segmentation result. (**a**) Color image-(**b**) Masked color pads’ image.

The next step is to filter each *C_y_*, *C_B_*, and *C_R_* channel. Table 1 shows these color thresholding data; Please refer to Figure 6a,b for the result of color based segmentation.

Each image convex hull is then extracted from each frame, and the best convex hull is cited as the smallest convex set that contains all the region’s pixels in the Euclidean plane [38,39,40].

(3) $$X_{k}^{i}=\left(X_{k-1}⊗B^{i}\right)\cup A\;i=1,2,3,4\;\mathrm{and}\;k=1,2,3,\ldots$$

(4) $$X_{0}^{i}=A\;\mathrm{and}\;D^{l}=X_{\mathrm{con}\;}^{i}$$

(5) $$X_{k}^{i}=X_{k-1}^{i}$$

(6) $$C(A)=\overset{4}{\underset{i=1}{\cup }}D^{i}$$

where *B^i^* and *X_k_* are the structuring elements of the binary image and the set of convex sets, respectively. In Equation (4), the subscript “con” denotes the convergence that satisfies Equation (5). Finally, considering Equation (6), the union of all the subsets of the convex set is the binary convex of all the image blobs.

Next, the centroid of each color pad is extracted by the following relationship:

(7) $$\{\begin{array}{l} \vartheta x=\frac{1}{N}\sum_{i}^{N}x_{i}\\ \vartheta y=\frac{1}{N}\sum_{i}^{N}y_{i} \end{array}$$

where *N* is the number of the binary image points [41].

Finally, all the collected centroid points and range data, containing close to 120 points, are stored, and the lookup table is made from this data. To project the data that are not included in the table, data is interpolated through the Piecewise Cubic Hermite Interpolating Polynomial (PCHIP). PCHIP is a very rapid and efficient algorithm and takes less than 0.03 s to calculate data interpolation.

To obtain $f_{k}^{\prime }$ at the $x_{k}$ point via the PCHIP algorithm:

Let us consider $h_{k}=x_{k+1}-x_{k},\;\mathrm{and}\;d_{k}=\left(y_{k+1}-y_{k}\right)/h_{k}$ as the slope between two interpolating points $x_{k+1},x_{k}$. The slope of the point between them has the Equation (9) as indicated.

(8) $$f_{k}^{\prime }=\{\begin{array}{c} \frac{\left(w_{1}+w_{2}\right)}{\left(\frac{w_{1}}{d_{k-1}}+\frac{w_{2}}{d_{k}}\right)}\\ 0 \end{array}\begin{array}{c} d_{k},d_{k-1}\neq 0\;\&\;Sign(d_{k})\neq Sign(d_{k-1})\\ d_{k},d_{k-1}=0\;\&\;Sign(d_{k})=Sign(d_{k-1}) \end{array}$$

(9) $$sign(x)=\{\begin{array}{c} 1\\ 0\\ -1 \end{array}\begin{array}{c} x>0\\ x=0\\ x<0 \end{array}$$

(10) $$h_{k}=x_{k+1}-x_{k},$$

(11) $$d_{k}=\left(y_{k+1}-y_{k}\right)/h_{k}$$

where *Sign* is the signal function [42]. In Equation (11), $d_{k}$ is the slope between two points, $x_{k+1}$, and $x_{k}$. Finally, a one-sided scheme is used to calculate the end slope of the interpolating shape [43].

### 2.3. Image Range Acquisition

**Figure 7:**
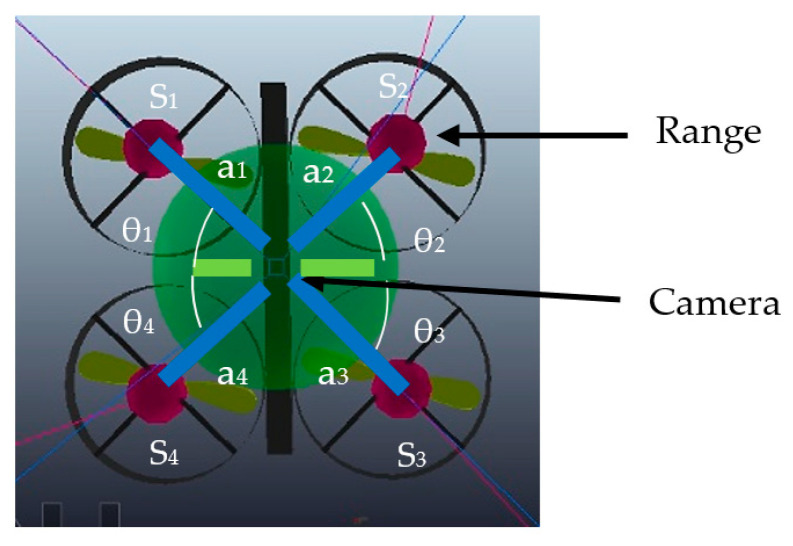
Range sensor positions and angles w.r.t the camera.

In this experiment, the image acquisition device is a monocular camera attached to the drone and which has the same coordinate frame as the drone, but in the downward direction. The resolution of the camera is 512 × 512 pixels. In addition, as depicted in Figure 7, there are four range sensors located in the same plane as the camera. There is a translational shift for these four range sensors. The coordinate system for the sensors is:

(12) $$\begin{array}{l} {\mathrm{Ps}}_{1}=[\;0\;0\;0];\\ {\mathrm{Ps}}_{2}{=[a}_{1}\times \cos(\theta_{1}{)+a}_{2}\times \cos(\theta_{2})\;0\;0];\\ {\mathrm{Ps}}_{3}{=[a}_{2}\times \cos(\theta_{2}{)+a}_{3}\times \cos(\theta_{3}{)\;a}_{2}\times \sin(\theta_{2}{)+a}_{3}\times \sin(\theta_{3})\;0];\\ {\mathrm{Ps}}_{4}{=[\;0\;a}_{1}\times \cos(\theta_{1}{)+a}_{4}\times \cos(\theta_{4})\;0]; \end{array}$$

where Ps_1_, Ps_2_, Ps_3,_ and Ps_4_, are the sensors’ locations in the camera frame, with the origin of the first sensor. Also, a*_i_* are the distances of the sensors from the camera and $\theta_{i}$ are assumed to be nearly 45°.

Figure 7 illustrates these angles and distances for the designed in Equation (12). As can be seen, each sensor is located at the same distance, presented in dark blue line with the magnitude of 0.1 m, from the camera and at the same angle magnitude, depicted in white curve with 45°, with respect to the camera’s axes.

### 2.4. Calculate the Homography between the Pixels and the World Coordinates

**Figure 8:**
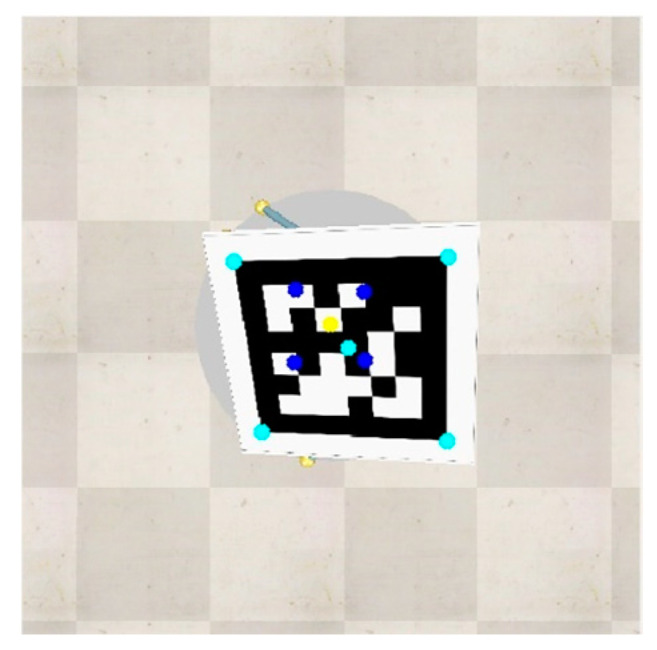
Touch points of the range sensor (blue), AprilTag center (cyan), AprilTag corners (cyan), and of the sensor unit principle point (yellow).

**Figure 9:**
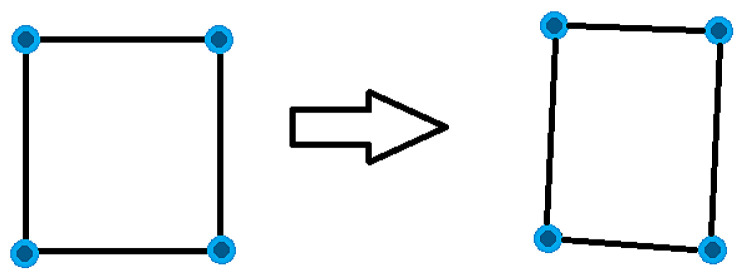
Transformation from world-corresponding points to sensor touch points in the image plane.

This step calculates the relation between an image pixel and the corresponding world coordinate points. The projective transformation has eight degrees of freedom [44]. Figure 8 shows the range sensor touch points, which are calculated through camera range sensor calibration (refer to Section 2.2), in dark blue. These are transformed by projective transformation, as illustrated in Figure 9 in image plane.

**Figure 10:**
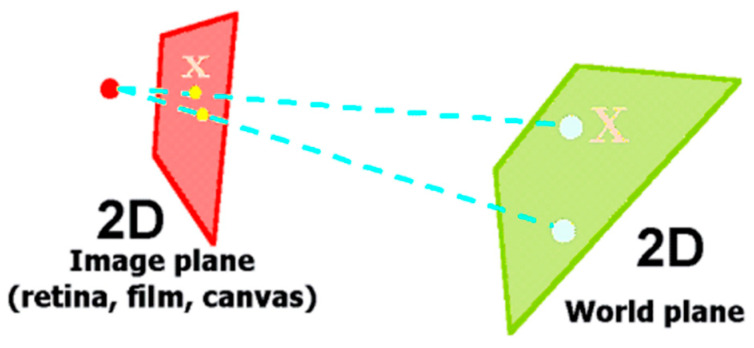
Image plane, red plane, and world plane, in light green, relations by homography.

Figure 10 demonstrates the relationship of a 2D image plane, presented in red and in left, and its projected 2D world plane, depicted in light green and in right.

The homogeneous coordinate system is used for this mapping with the following relation [45,46,47,48]:

(13) $$u^{\prime }=Hu$$

(14) $$\left[\begin{array}{c} x_{1}\\ y_{1}\\ 1 \end{array}\right]=H\left[\begin{array}{c} x_{2}\\ y_{2}\\ 1 \end{array}\right]=\left[\begin{array}{ccc} h_{11} & h_{12} & h_{13}\\ h_{21} & h_{22} & h_{23}\\ h_{31} & h_{32} & h_{33} \end{array}\right]\left[\begin{array}{c} x_{2}\\ y_{2}\\ 1 \end{array}\right]$$

in which it is assumed that $h_{22}$ is taken as one, hence there are 8 variables, the 8 Degrees of Freedom (DoF), in the homography matrix, denoted by H in Equation (14) [49].

(15) $$x_{1}=\frac{h_{11}x_{2}+h_{12}y_{2}+h_{13}}{h_{31}x_{2}+h_{32}y_{2}+h_{33}}$$

(16) $$y_{1}=\frac{h_{21}x_{2}+h_{22}y_{2}+h_{23}}{h_{31}x_{2}+h_{32}y_{2}+h_{33}}$$

In Equations (15) and (16), $x_{1}$, $y_{1}$, $x_{2}$, and $y_{2}$ are corresponding points that undergo projective transformation. To solve Equations (15) and (16) at least four points are required. The order of the points is vitally important to achieve a meticulous transformation. The world coordinates of the sensor are as stated in Equation (12) with the order as given below in Equation (17):

(17) $${\mathrm{PSWorld}}_{\mathrm{xy}}=\left[\begin{array}{c} {\mathrm{ps}}_{1\mathrm{xy}}\\ {\mathrm{ps}}_{2\mathrm{xy}}\\ {\mathrm{ps}}_{4\mathrm{xy}}\\ {\mathrm{ps}}_{3\mathrm{xy}} \end{array}\right]$$

(18) $${\mathrm{ProjectedPoints}}_{vu}=\left[\begin{array}{c} Pxl{\left(BLUE\right)}_{vu}\\ Pxl{\left(\mathrm{GREEN}\right)}_{vu}\\ Pxl{\left(\mathrm{RED}\right)}_{vu}\\ Pxl{\left(\mathrm{YELLOW}\right)}_{vu} \end{array}\right]$$

where PSWorld_xy_ are the 2D world points with *X*-*Y* coordinates only, and ${\mathrm{ProjectedPoints}}_{vu}$ are the projected points in the pixel plane that is flipped in the *u* and *v* directions.

### 2.5. Mapping from Apriltag Pixels to World Coordinates by Inverse Homography

Now that we have the relation between the world plane and its corresponding image, by using inverse planar homography, all points on the 2D image plane can be mapped to 2D world points, presented in worlds’ *X* and *Y* coordinates. This can be conducted easily through inverse homography of the projective transform found in the previous subsection and by the following Equation (19) [50,51,52]

(19) $$u_{l}=H^{-1}u^{\prime }$$

where $u_{l}$ represents the 2D coordinates of the landmark, here AprilTag, with four known points.

### 2.6. Calculation of Points’ Altitude in the Camera Frame

Since there are four range sensors mounted on the quadrotor’s arm, it is easy to find the landing platform planar Equation (20) [53,54,55]:

(20) $$\hat{n}=(pst_{1}-pst_{2})\times (pst_{2}-pst_{3})$$

(21) $$pst=[\begin{array}{cc} ps_{xy} & d \end{array}]$$

where pst represents the sensors’ touch coordinates in the camera frame. In addition, $\hat{n}$ and $ps_{xy}$ denote the planar normal vector of the landing surface and the sensor coordinates in the X and Y directions, respectively.

Having the planar Equation (20) and the landmark’s 2D coordinates, $u_{l}$ in the Equation (19), it is convenient to find the *Z* coordinates of the points on the landing surface by means of Equation (22).

(22) $$u_{l_{Z}}=\frac{\left(d-{\hat{n}}_{1}\cdot u_{l_{X}}-{\hat{n}}_{2}\cdot u_{l_{Y}}\right)}{{\hat{n}}_{3}}$$

### 2.7. Estimate Rigid Body Transformation

At this stage, all the point coordinates of the landmark are known. Hence, the landmark is considered as a rigid body in the real world, and the transformation between the real-world coordinates and the calculated points is in the frame of the camera. The following Equations (23) and (24) are used to find the coordinates of the transformed points [56].

(23) $$x^{\prime }=Rx+t=R_{X}R_{Y}R_{Z}x+t$$

(24) $$t=\left[\begin{array}{c} t_{X}\\ t_{Y}\\ t_{Z} \end{array}\right]$$

In Equation (23), *R* is the rotation matrix and is expressed in Euler-angle order (pitch, roll and yaw) in “*ZYX*” order, with the angles expressed as in Equation (25). Moreover, variable *t* is the translational transformation in the *X*, *Y*, and *Z* directions, as stated in Equation (24). Euler angle vector, which are ϕ (roll), θ (pitch), and ψ (yaw), of the landing surface is indicated in Equation 25.

(25) $$Angle(R)=\left[\begin{array}{c} \phi \\ \theta \\ \psi \end{array}\right]$$

### 2.8. Transformation of the Coordinates

Considering the pixel coordinate flip in the homography calculation due to the order convention used in the CoppeliaSim simulator, a transformation would be indispensable to calculate the final corrected transformation.

(26) $$R_{cor}=R_{x}(-\phi )\cdot R_{y}(-\psi )\cdot R_{z}(\theta )$$

Using Equation (26), the corrected rotational components are calculated in Tait–Bryan angles with the order of ‘*xyz*’, with the result expressed in Euler-angle order (*ZYX*). Additionally, the translation is negated in the *X*-direction.

(27) $$t_{cor}=\left[\begin{array}{c} -t_{X}\\ t_{Y}\\ t_{Z} \end{array}\right]$$

**Figure 11:**
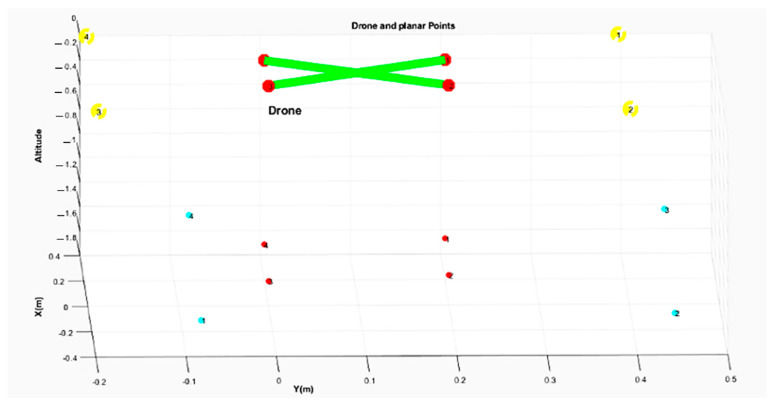
Estimation of the real-world 3D points in the camera frame.

The results of this stage are the 3D coordinates of the landmark in the real world. Figure 11 depicts the correction of the frame along with its calculated points, and calculated touch points through calibration process in the image coordinate. The cyan and red dots are the landmark corners and sensors’ touch points, respectively, on the surface, which are in a lower altitude than that of the sensor-camera platform. The drone-mounted sensors are depicted at zero altitude. Finally, yellow dots indicate the stable landmark coordinates in the camera frame.

## 3. Experimental Design

The settings of the experiment and software implementations are elaborated in the following sections.

### 3.1. Test Platform

**Figure 12:**
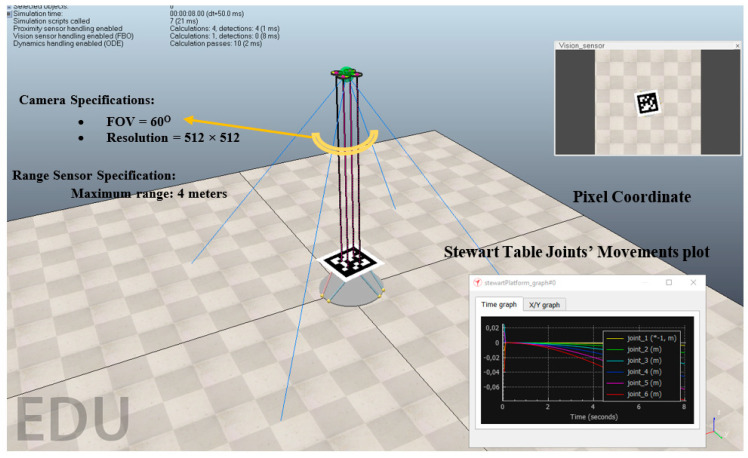
Experiment bench for the assessment of the technique along with Coordinate system used.

Figure 12 illustrates the overview of the test bench. The platform has dynamic translation and rotation. In addition, the AprilTag frame and the landing platform frame are assumed to be the same attitude. It is assumed that there is a motionless flying quadrotor and variable attitude landing platform.

### 3.2. Software Implementation

**Figure 13:**
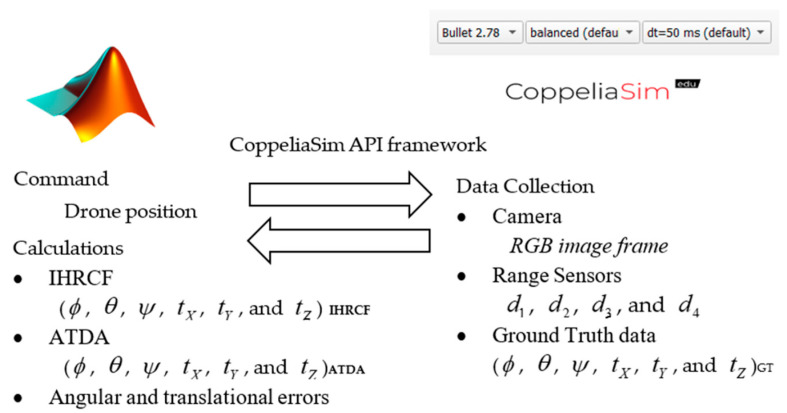
Software-in-the-Loop (SIL) data transmission and the connection protocol.

The simulator used in this experiment is called CoppeliaSim which has the potential to be controlled through the remote Application Programming Interface (API) using CoppeliaSim API framework and to be sent data in real time to a variety of software including MATLAB, Python, etc. From Figure 13, MATLAB software has the control of the drone dynamics, while sensors and camera data are sent back to MATLAB for the calculations. Ground truth data, including Euler angles and translational movements data of the landing platform are sent back to MATLAB for the evaluation of the proposing technique and ATDA for a comparative evaluation. The simulation engine employed were Bullet physic engine version 2.78 with the simulation time step of 50 ms and balanced speed. *d*_1_, *d*_2_, *d*_3_, and *d*_4_ are ranges measured through four range sensors. *ϕ*, *θ*, *ψ*, *t_X_*, *t_Y_*, and *t_Z_* are roll, pitch, yaw, translation in *X*, translation in *Y*, and translation in *Z*, for landing platform, respectively; The subscript IHRCF, ATDA, and GT indicate the type of data in Figure 13.

## 4. Results and Discussion

**Figure 14:**
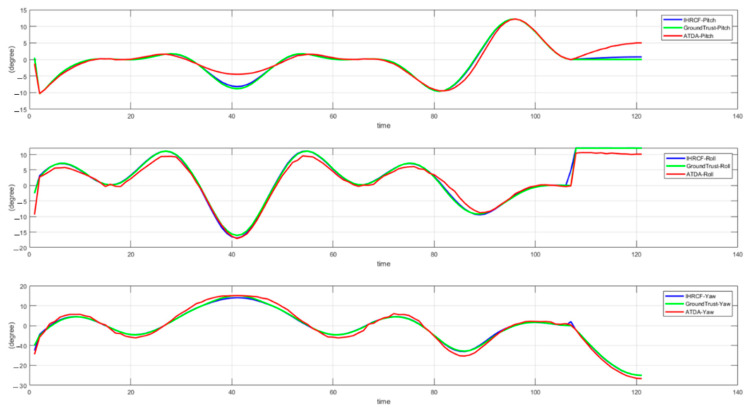
Euler angles, namely roll-pitch-yaw for the Stewart platform using IHRCF, GT, and ATDA.

**Figure 15:**
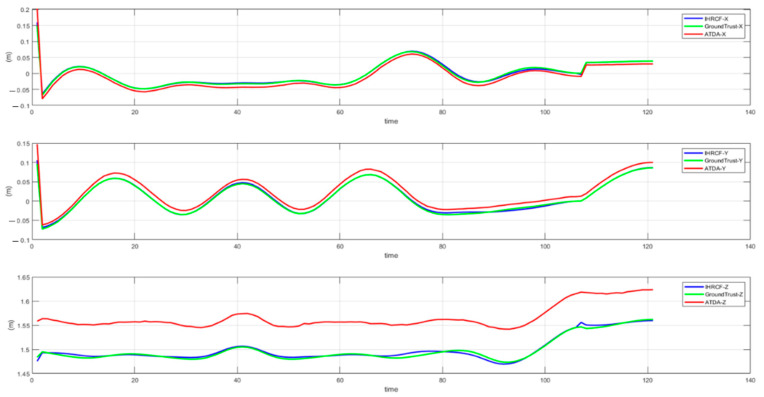
Translational results in *X*, *Y*, and *Z* directions for the Stewart platform using IHRCF, GT, and ATDA.

**Figure 16:**
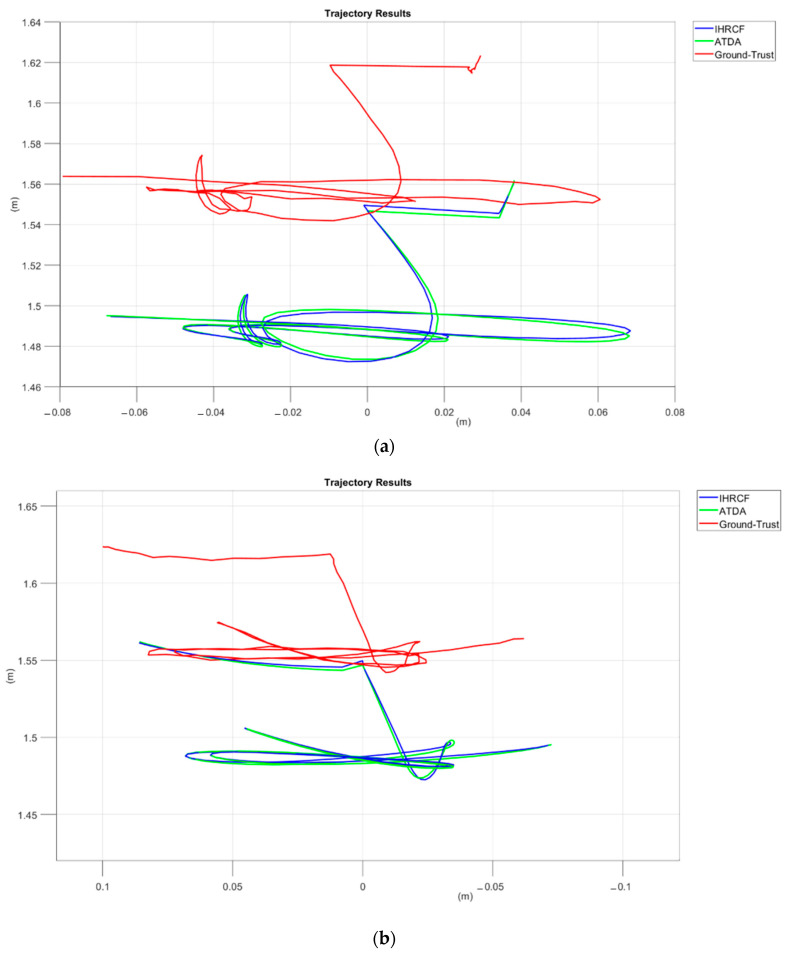

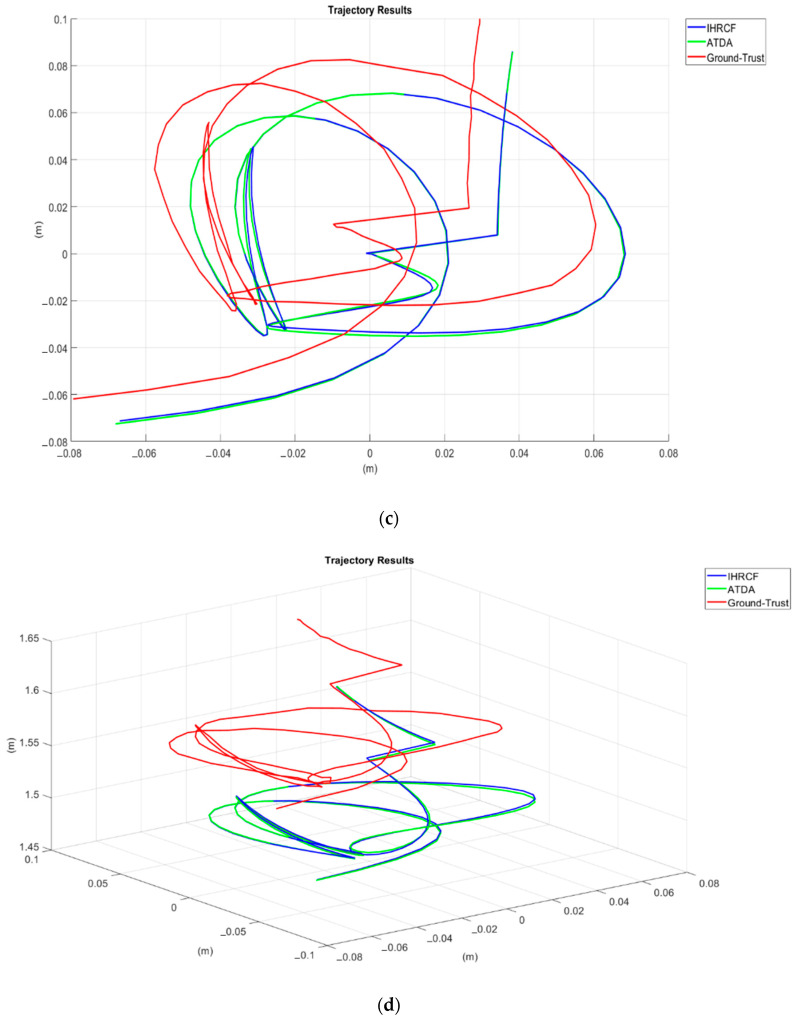
Plots of 3D trajectories of the Stewart platform using the IHRCF, ATDA base pose estimation and the GT, with the *X*-*Z* view, *Y*-*Z* view, *X*-*Y* view, and 3D trajectory displayed in (**a**–**d**), respectively.

The IHRCF pose estimation conducted in this research is examined in its translational and rotational components. Figure 14 shows the angular comparison of the IHRCF, Ground Truth, and ATDA approaches in the simulation environment, presented in blue, green, and red, respectively. The results confirm a robust tracking of the ground truth by the IHCRF, and by the ATDA for roll and pitch. From Figure 15, IHRCF proved a significant improvement in the altitude estimation, while it slightly ameliorated the estimation in *X* and *Y* directions. ATDA shows major inaccuracy for *Z* direction distance calculation. Figure 16 illustrates the translational results of the IHRCF (in blue) compared to those of the ATDA, in red, and the Ground truth (GT), in green. The IHRCF offers a better tracking of the GT than the AprilTag algorithm. At 40 and 120 s ATDA technique proved slight inaccuracy, while IHRCF rectified the error using range sensor data.

Figure 16 depicts the IHRCF trajectory plots (in blue) of the descending *XZ*, *YZ*, *XY*, and *XYZ* surfaces in (a–d), respectively, compared to those of the AprilTag/camera pose detection technique (in red) and ground truth (in green). The pose estimation errors are portrayed next.

**Figure 17:**
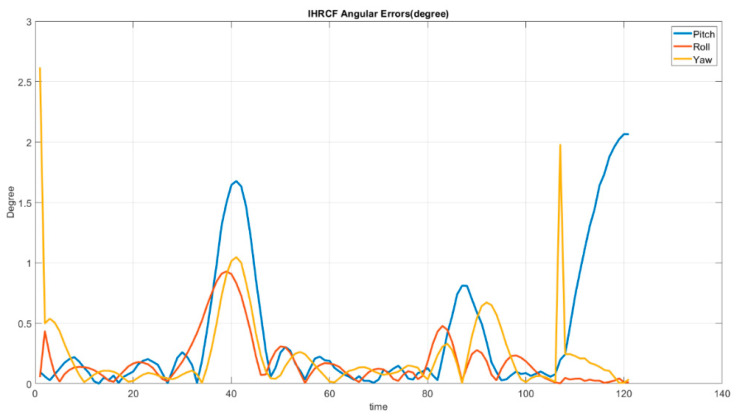
IHRCF-based Stewart platform Euler angles estimation error along the *X* (blue), *Y* (red), and *Z* (yellow) axes.

Figure 17 shows the IHRCF transitional errors, revealing that they do not exceed 0.04 m. Disregarding initial errors, the proposing technique pose estimation error stays below 0.01 m, with the maximum error in the *Z* direction at around 110 s.

**Figure 18:**
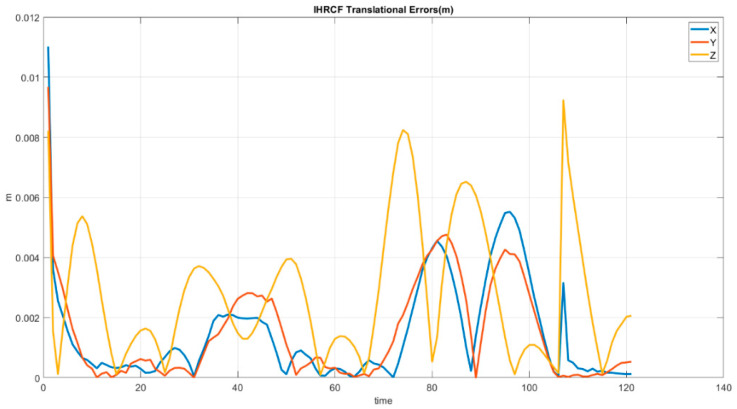
IHRCF translational pose estimation errors for the Stewart platform along the *X* (blue), *Y* (red), and *Z* (yellow) axes.

Figure 17 shows the IHRCF Euler angle estimations errors along the *X*, *Y* and *Z* axes, while Figure 18 demonstrates the IHRCF translational errors. Regarding Figure 17, excluding initial inaccuracy of the IHRCF, maximum error belongs to pitch calculation at 120 s and in the remaining period the errors are beneath 2 degrees. Considering Figure 18, the maximum error is, for *Z* direction, in 110 s with 0.009 m and in the rest of the periods the errors stayed less than 0.0085 in all axes.

**Figure 19:**
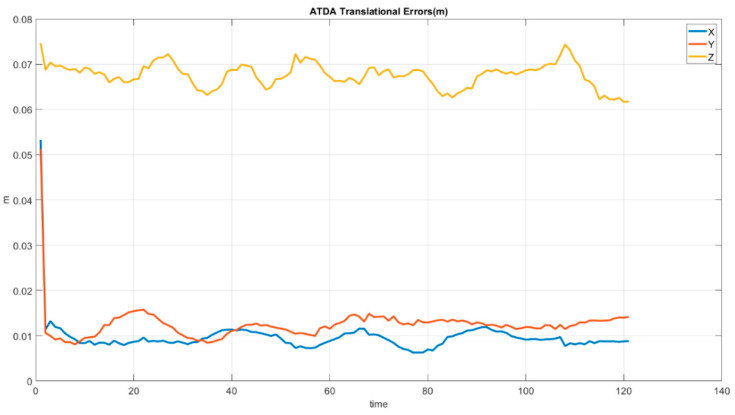
ATDA base Stewart platform translational estimation error along the *X* (blue), *Y* (red), and *Z* (yellow) axes.

**Figure 20:**
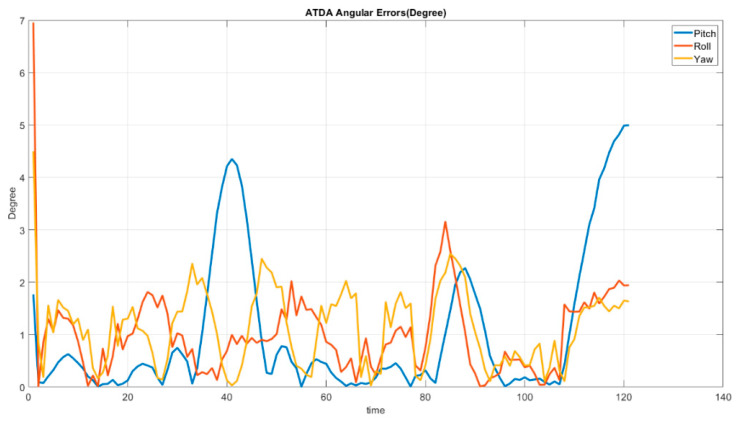
ATDA base Stewart platform angular estimation error along the *X* (blue), *Y* (red), and *Z* (yellow) axes.

As it can be inferred from Figure 19 the AprilTag detection algorithm has a significant inaccuracy in the *Z*-direction calculation, while the rest of the directions’ error is below 0.02 m. Figure 20 illustrates the striking angular estimation error at 120 s with 5 degrees and at 4 s with approximately 4.3 degrees in pitch. In addition, roll inaccuracy happens at 83 s with 3 degrees. However, in the rest of the simulation error remained less than 3 degrees.

As shown in Figure 19 and Figure 20, the ATDA demonstrates high angular and translational estimation errors. Equation (28) shows Mean Absolute Error (MAE) calculation over the whole simulation process.

(28) $$\mathrm{MAE}=\frac{\sum_{t=t_{1}}^{t_{n}}\left|y_{t}-{\tilde{y}}_{t}\right|}{n}=\frac{\sum_{t=t_{1}}^{t_{n}}\left|e_{t}\right|}{n}$$

*y_t_*, ${\tilde{y}}_{t}$, and *e_t_* are ground truth, estimated pose, and error at time *t* respectively. The MAEs of the proposed IHRCF system are summarized and compared to those of the ATDA in Table 2.

Table 2 shows the improved error values in both angular terms and translational estimations realized by the IHRCF in relation to those of the ATDA. The estimation errors for the translational results’ peak are at close to 0.073 m and 0.0085 m for the ATDA, in the *Z* direction, and for the IHRCF, in the *X* direction, respectively. In addition, the ATDA had maximum angular errors of 5 degrees for the pitch angle, while the angular error peak for IHRCF was 2 degrees for the yaw angle.

## 5. Conclusions and Future Work

The prime aim of the current research was to develop a process that can support emergency landing systems in highly complex contexts, such as drone marine deck landing. The proposed IHCFR algorithm addresses the problem of pose estimation. This research showed that single camera-based pose estimation is not adequate to calculate the attitude of a descending platform, as the camera model is not obtained correctly via the camera calibration process. A precise intrinsic camera parameter is vitally important to find the real-world coordinates of the landmark. The IHCFR algorithm performed pose estimation more accurately than the ATDA, but it requires four accurate range sensors. The algorithm employs range sensors and proposing camera-ToF calibration data to calculate planar homography. Then the inverse of homography, normal vector found by ranges data, and pixel coordinate of the corners are used to calculate the world coordinate of the corners for the AprilTag. A rigid body estimation method is used to find rotational and translational behavior of the landing platform. Finally, a transformation correction of the coordinate from simulator allocated coordinates is calculated. Overall, the IHRCF algorithm outperformed the ATDA in these experiments in rotational and translational pose estimation. An interesting aspect of this proposed algorithm is that it is independent of the camera calibration process and thus does not have the deficiencies of inaccurate camera calibration. As a future work, it is recommended that Inertial Measurement Units (IMUs) are included in the landing system design to address its the weaknesses, such as sensor interaction noise among the ToF range sensors.

## Figures and Tables

**Table 1:** YCBCR base color thresholding for blue, green, yellow, and red.

Color	Channel *Y*	Channel *C_B_*	Channel *C_R_*
Blue	0 ≤ Y ≤ 165	139 ≤ *C_B_* ≤ 255	0 ≤ *C_R_* ≤ 255
Green	0 ≤ *Y* ≤ 255	0 ≤ *C_B_* ≤ 155	0 ≤ *C_R_* ≤ 90
Yellow	103 ≤ *Y*≤ 255	0 ≤ *C_B_* ≤ 95	0 ≤ *C_R_* ≤ 255
Red	0 ≤ *Y* ≤ 160	0 ≤ *C_B_* ≤ 255	167 ≤ *C_R_* ≤ 255

**Table 2:** Mean absolute error comparison of AprilTag and IHRCEFcalibration algorithms.

Absolute Error.	ATDA			IHRCF		
Translations (m)	0.0162	0.0134	0.0697	0.0035	0.0039	0.0041
Angles (degree)	2.9843	1.657	1.7743	0.98	1.3731	1.180

## Data Availability

Not applicable.
